# Development of a Novel *Aedes aegypti* Repellent: An Integrated Approach Combining In Silico and Bioassays

**DOI:** 10.1002/cbdv.202501251

**Published:** 2025-12-03

**Authors:** Moysés Fagundes de Araujo Neto, Jânio Rodrigo de Jesus Santos, Ana Luiza Pontes Silva de Oliveira, Antonio Wanderson Vieira Gois, Ingrid Bernardes Santana Martins, Vitor Barbanti Pereira Leite, Adriano Figueiredo Monte Alegre, Jairo Torres Magalhães Junior, Franco Henrique Andrade Leite

**Affiliations:** ^1^ Laboratório de Quimioinformática e Avaliação Biológica Departamento de Saúde Universidade Estadual de Feira de Santana Feira de Santana Bahia Brazil; ^2^ Instituto de Ciências da Saúde Universidade Federal da Bahia Salvador Bahia Brazil; ^3^ Centro Multidisciplinar Departamento de Saúde Universidade Federal do Oeste da Bahia Barreiras Bahia Brazil; ^4^ Departamento De Física—IBILCE Universidade Estadual Paulista São Paulo Brazil

**Keywords:** *Aedes aegypti*, constant pH molecular dynamics (CpHMD), odorant‐binding protein, repellence assays, virtual screening

## Abstract

*Aedes aegypti* is the most important vector of arboviruses, which are responsible for 3.9 billion cases worldwide. This mosquito seeks a host using its olfactory system, where the odorant‐binding protein (OBP) in *A. aegypti* (*Aaeg*OBP1) is essential in sensory communication and is the first filter in odorant selection, which makes this target promising for the development of new repellents. In order to help the *Aaeg*OBP1 modulator prioritization, a validated in silico methodology was employed, which enabled the selection of a potential repellent. ZINC71773878 showed a dependency relationship between dosage and repellency activity in bioassays. At the highest concentration used (3000 µmol/100 µL), the ZINC71773878 showed a higher repellency rate than the positive control (*N,N*‐diethyl‐3‐methylbenzamide [DEET]). The constant pH molecular dynamics (CpHMD) approach employed was able to elucidate the energetic contributions and the effects of different pH on *Aaeg*OBP1–ZINC71773878 complex dissociation at pH = 4.0. ZINC71773878 must be prioritized for further trials aiming to develop a new product with repellent activity that is safer and more efficient than the available commercial repellents in Brazil.

## Graphical abstract

OBP1 plays an essential role in the behavior response of *Aedes aegypti*. The computational approaches employed herein suggest a compound with repellency activity in the ZINC database, which was confirmed by bioassays.

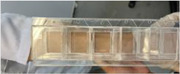

## 1 Introduction

Brazilian public health history is marked by infectious diseases caused by the urban and peri‐urban proliferation of *Aedes aegypti* mosquitoes, which is considered the main endemic disease vector in the world. Among the diseases that *A. aegypti* can transmit, the arboviruses, such as dengue virus (DENV), Zika virus (ZIKV), chikungunya virus (CHIKV), and yellow fever virus (YFV), which infect 3.9 billion people and are responsible for 600 thousand deaths in world, can be highlighted [1, 2].

The highly populated urban environment has population dynamics that generate ideal habitats for *A. aegypti* proliferation; together with the lack of effective strategies to combat the mosquito, morbidity forces of infectious agents, and the high adaptability of the mosquito, it makes it a very difficult vector to eradicate [3].

*A. aegypti* mosquitoes use their chemosensory systems, along with other sensory modalities, to identify volatile organic compounds (VOCs) to locate their host and find blood, which is necessary for egg production [4, 5]. This sensory perception is initiated by the presentation of VOC to neuroreceptors by odorant‐binding protein (OBP), which takes these hydrophobic molecules through the hydrophilic lymph to the transmembrane receptors in the neurons [6], playing a critical step in the first stage of olfactory signal transmission.

OBPs play an essential role as the first filter of sensitivity and odor selection in the olfactory system of insects [7, 8]. Among 66 OBPs in the *A. aegypti* genome, OBP1 is the most expressed in female mosquitoes, being the main candidate to explain mosquito host selection behavior [9].

The therapeutic treatment of *A. aegypti* arboviruses is symptomatic, where there is no approved drug capable of combating the arbovirus. On the other hand, there is a vaccinal scheme for preventive control of the DENV, but it does not guarantee efficacy due to the different viral strains, which makes these alternatives not useful. This way, the vector control has become a promising strategy for arbovirus control, such as the use of larvicides, pupacides, adulticides, and repellents [10].

The first three strategies are based on the use of chemicals (e.g., pyrethroids) integrated into environmental management programs. However, the continued use of these strategies has toxic effects on humans (e.g., leukemia, neurological disorders, and mucosal irritations) and the environment. Additionally, the continued exposure can lead to resistance to *A. aegypti*, which can favor an increase in the mosquito population and, consequently, the number of arbovirus cases. On the other hand, the use of repellents is based on the use of synthetic compounds inspired by natural products (NPs) (e.g., *N*,*N*‐diethyl‐3‐methylbenzamide—DEET, Icaridin, and IR3535) or essential oils, which modify the mosquitoes’ behavior. Despite the short duration of action of currently available repellents (approximately 5 h), their use has fewer adverse effects on humans compared to other vector control strategies [10].

The mosquito olfactory system plays an important role in *A. aegypti* behavior because it allows the selection of host and oviposition sites [11]. Thus, the identification of new olfactory modulators against *A. aegypti* OBP1 (*Aaeg*OBP1) could interfere with the sensory pathway of mosquitoes, resulting in the blockade of chemical signal transduction and, thus, generate a repellent behavior in mosquitoes. Additionally, OBP1 is highly expressed in several female mosquito species (e.g., *Anopheles gambiae*, *Anopheles funestus*, and *Culex quinquefasciatus*) [6].

Computational approaches can be employed to prioritize potential modulators with stereo‐electronic features and binding affinity to *Aaeg*OBP1 (e.g., pharmacophore models) and the same binding mode of commercial repellents (e.g., molecular docking and molecular dynamics [MD] integration) [12, 13]. Thus, it helps in the prioritization of potential compounds capable of binding to *Aaeg*OBP1 and generating repellent behavior in the mosquito for biological assays.

This way, the exposure of vector to controlled concentrations of a compound (e.g., repellency tests) allows the observation of the compound's ability to modulate the behavior of *A. aegypti* [12]. Thus, the use of hierarchical virtual screening together with MD studies and repellency assays can help in the identification of new compounds with repellency properties against *A. aegypti*.

Additionally, the constant pH MD (CpHMD) approach was employed to gain insights into the pH‐dependent mechanism of *Aaeg*OBP1, particularly in the context of odorant presentation (pH = 8.0) and release (pH = 4.0). This approach contributes to a better understanding of the molecular basis underlying mosquito behavior modulation. Thus, this study aimed at the selection of compounds with repellent activity against *A. aegypti* through computational approaches integrated with repellency assays and elucidated the pH‐dependent mechanism of *Aaeg*OBP1 by CpHMD simulations.

## 2 Results and Discussion

### 2.1 Pharmacophore Model Build and Evaluation

Pharmacophore‐based virtual screening plays an essential role in the prioritization of compounds with the same stereo‐electronic features of commercial repellents. This way, the definition of stereo‐electronic features that modulate the mosquito behavior is directly related to potent compounds with repellency activity in the dataset employed on pharmacophore hypothesis construction [4].

One challenge to pharmacophore model construction is the chemical diversity and high structural flexibility of *Aaeg*OBP1 modulators employed in the dataset, which can lead to energetic penalties during three‐dimensional (3D) alignment [9, 14]. This way, Genetic Algorithm with Linear Assignment for Hypermolecular Alignment of Datasets (GALAHAD) was employed to generate 10 pharmacophore models from a set of compounds (training set) with affinity to *Agam*OBP1 [6] based on high structural similarity in comparison with *Aaeg*OBP1.

Among the 10 pharmacophore models that were built, three were discarded on the basis of strain energy criteria (<100.0 kcal/mol) [15] (Table S1).

In order to evaluate the remaining pharmacophore models, the Pareto index (Energy, Sterics, H_bond, and Mol_qry) shows that the remaining pharmacophore models were statistically equivalent (Pareto = 0) and, thus, there is no dominant statistical parameter, and it was not possible to discard none of them [16].

Another computational approach that can be employed to discriminate the best pharmacophore model is through the ability to differentiate active compounds from false positives (decoys) [17, 18]. This way, the best pharmacophore model will have the highest sensitivity to identify active compounds according to receiver operating characteristic (ROC) curve analysis and area under the curve (AUC) value [19].

A dataset with 18 *Agam*OBP1 modulators (IC_50_ < 20 µM) and 900 decoys was used to build ROC curves and calculate AUCs (AUC–ROC) using the superposition value of compounds in each model (QFIT value; 0–100). An AUC equal to 1.0 would be found in a model with the capacity to recognize all actives before decoys, and an AUC less than 0.5 would be associated with models worse than a random selection. However, an AUC equal to 0.70 is associated to moderate predictive ability [19]. Thus, pharmacophore model 03 (AUC = 0.70) was selected as the most reliable pharmacophore of *Agam*OBP1 (Figure S1).

The best pharmacophore hypermolecule has two hydrophobic centers (HY) and one hydrogen bond donor (HBD) (Figure S2). In addition, SAR studies have shown that the presence of hydrophobic centers and donor groups in the chemical structure of repellent compounds is an essential requirement present in molecules with affinity to *Aaeg*OBP1 [9, 20]. In order to characterize potent repellents with the pharmacophore, a potent compound was aligned to Model 3 (Figure S2A). In addition, a commercially available repellent (Icaridin) was aligned to the model, where it was possible to visualize the total structure superposition in pharmacophore features (Figure S2B).

This way, the pharmacophore model 03 can reproduce the requirements of *Aaeg*OBP1 and commercially available repellents and thus is useful to prioritize potential compounds with *Aaeg*OBP1 affinity.

### 2.2 Molecular Docking Studies

Molecular docking approaches are widely used to predict the binding affinity of compounds to *Aaeg*OPB1 [4, 20]. When crystallographic ligand information is unavailable for the macromolecule, non‐native crystallographic ligands can be employed through cross‐docking methodology [21, 22]. Thus, the comparison of *Agam*OBP1 (PDB: 3N7H), as a reference control, and *Aaeg*OBP1 (PDB: 3K1E) sequences showed 82.1% of sequential identity between the two macromolecules. This comparison facilitated the definition of the active site and the validation of docking studies using DEET (the crystallographic ligand of AgamOBP1) within the AaegOBP1 active site. The root‐mean‐square deviation (RMSD) of the residues was calculated using the Biopolymer [23] program to assess atomic variations in the structures and their cavities, revealing no significant atomic differences between *Aaeg*OBP1 and *Agam*OBP1 (RMSD = 1.03 Å). The positioning of residues in the active sites of both proteins was confirmed to be the same. Given the high sequence identity, we inferred that *Agam*OBP1 and *Aaeg*OBP1 exhibit similar 3D conformations, thereby justifying the cross‐docking approach for molecular docking validation (Figure S3).

The GOLD program was employed for docking studies, utilizing a genetic algorithm to account for the flexibility of compounds with repellency activity and the *Aaeg*OBP1 active site in a molecular docking study and predicting the most stable conformer of each compound [10]. Additionally, the GOLD program can score the conformers’ affinity in the active site by the definition of force field and intermolecular interactions (e.g., GoldSCORE), enhancing the potential to reproduce biological data from a training set (e.g., ChemPLP and ChemSCORE) or establish statistical potentials based on atomic contact frequencies in ligand–macromolecule complex (e.g., ASP) [24].

The effectiveness of the scoring functions was evaluated by comparing the best pose obtained by the GOLD program and the DEET pose from the crystallographic structure [25]. A satisfactory solution is indicated when the RMSD of atomic positions between the two poses is less than 2.0 Å. This approach is also straightforward to interpret, demonstrating the program's ability to identify the correct orientation.

Among the scoring functions implemented in the GOLD program, three showed RMSD values exceeding 2.0 Å (RMSD_ASP_ = 8.243 Å; RMSD_ChemSCORE_ = 8.100 Å; RMSD_GoldSCORE_ = 2.92 Å) and were therefore excluded from further analysis. In contrast, ChemPLP yielded an RMSD value below 2.0 Å (RMSD = 0.703 Å; Figure S4).

ChemPLP, the first scoring function implemented in GOLD, considers both external (hydrogen bonding and van der Waals [VdW] forces) and internal (VdW forces and torsional energy) energies for predicting compound affinity in the active site [24]. Consequently, the ChemPLP fitness function was employed in the molecular docking study based on the RMSD value (RMSD = 0.703 Å).

### 2.3 Pharmacophore‐ and Docking‐Based Screening

Among 216 086 compounds from Sigma‐Aldrich (https://www.sigmaaldrich.com) and Biogenic databases, 129 279 compounds superposed to pharmacophore model 03 with partial stereo‐electronic requirements (0.13 < QFIT > 96.99). As a pharmacophore‐based virtual screening result, 1681 compounds (QFIT > 81.53) were prioritized with QFIT between 79.10 and 96.99 for molecular docking study.

Among prioritized compounds from docking‐based virtual screening, 1271 compounds made interactions in the *Aaeg*OBP1 active site and did not have steric penalties (ChemPLP > 0).

### 2.4 Physical–Chemical and Skin Sensitization Filtering

The physical–chemical requirements of compounds with *Aaeg*OBP1 affinity were predicted to prioritize compounds with the same volatile requirements as repellents. A total of 28 compounds capable of binding *Aaeg*OBP1 were used to measure the physical–chemical requirements [9]. In addition, the skin sensitization was carried out through the *pk*CSM online server as a primary skin irritation study, aiming at the commercial viabilization [26].

Five of 1271 compounds with *Aaeg*OBP1 affinity had a molecular weight (MW) less than 250 Da (MW < 250 Da); a polar surface area (PSA) between 60 and 101 (60 Å^2^ <PSA> 101 Å^2^); a partition coefficient between 1.536 and 3.127 (1.54 <cLog *P*> 3.13); less than five hydrogen bonding acceptors (HBA < 5); and less than two hydrogen bonding donors (HBD < 2) and were prioritized as potential compounds with *Aaeg*OBP1 affinity (Table 1).

**TABLE 1:** Physical–chemical prediction of molecules selected in the virtual screening step.

Molecule	MW	PSA	HBD	HBA	clog *P*	Skin sensitization	Eye corrosion	Respiratory toxicity
**ZINC380698**	219.27	68.01	2	3	2.23	No	0.0	0.93
**ZINC71773878**	228.25	61.17	0	3	1.91	No	0.02	0.77
**ZINC62702141**	244.25	72.80	0	4	1.68	No	0.04	0.59
**ZINC10483047**	230.22	72.80	0	4	1.75	No	0.05	0.63
**ZINC17917305**	222.25	41.46	2	1	2.63	No	0.01	0.82
**DEET**	191.13	20.31	0	2	2.128	No	0.16	0.82

*Note*: The IUPAC name and chemical structure are available in the Supporting Information section.

Abbreviations: clog *P* = octanol–water partition coefficient; HBA = hydrogen bond acceptor; HBD = hydrogen bond donor; MW = molecular weight (Da); PSA = polar surface area (Å^2^).

The in silico toxicological analysis of the prioritized compounds revealed promising safety profiles, particularly regarding skin sensitization. All predicted compounds—including the commercial repellent DEET—showed negative results for this endpoint. This finding is especially relevant for topical repellent applications, as the absence of skin sensitization potential reduces the risk of allergic reactions and contact dermatitis, thereby improving user safety [26]. The consistency of these negative predictions across all tested molecules provides a solid foundation for further formulation development.

With respect to eye corrosion, the predicted values were low for most compounds, ranging from 0.0 to 0.05, indicating minimal potential for ocular irritation. In contrast, DEET presented a higher predicted value (0.16), suggesting a comparatively greater risk for this endpoint. These results highlight the candidate compounds evaluated in this study as safer alternatives to DEET, particularly in formulations that may come into contact with the eyes or mucous membranes.

For respiratory toxicity, ZINC380698 (0.93) and ZINC17917305 (0.82) showed higher predicted values compared to DEET (0.82), suggesting a potential risk under inhalation exposure, especially in aerosol‐based applications. However, these values should be interpreted with caution due to the inherent limitations of predictive in silico models. Notably, compounds such as ZINC71773878 (0.77), ZINC62702141 (0.59), and ZINC10483047 (0.63) demonstrated comparatively lower predicted respiratory toxicity than DEET. These findings emphasize the importance of selecting appropriate dosage forms—such as lotions or gels—to minimize inhalation‐related adverse effects during repellent use.

In summary, the prioritized compounds demonstrated safety profiles comparable to or better than that of commercial repellents, particularly ZINC71773878, ZINC62702141, and ZINC10483047, which stand out as promising candidates for further development. In addition, safety and efficacy assays are required by current regulations for the approval of new repellents by regulatory agencies [26, 27].

## 3 Molecular Dynamics

Although the integration of pharmacophore‐ and docking‐based virtual screening is capable of prioritizing potential olfactory modulators, these approaches do not allow considering thermodynamic and biological factors that may affect the stability of the complexes and, consequently, the affinity of the compounds toward the biological target. Thus, the application of strategies that use the flexible ligand–macromolecule complex in a solvated environment is capable of providing information close to biological phenomena.

Additionally, short‐timescale MD simulations (on the order of tens of nanoseconds) can be effectively integrated into hierarchical virtual screening workflows as a strategic tool to refine and prioritize ligand candidates before proceeding to biological assays. In our study, the 40 ns MD simulation was not intended to provide exhaustive conformational sampling but rather to assess the initial stability and dynamic behavior of the OBP–ligand complex, thereby supporting the selection of the most promising compound for subsequent experimental validation. This approach has been widely adopted in structure‐based drug discovery to balance computational efficiency with predictive reliability, particularly when used in combination with docking and other in silico filters [22, 28].

**FIGURE 1:**
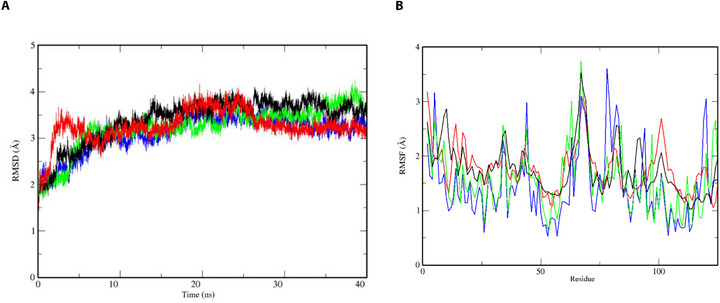
(A) Root‐mean‐square deviation of atomic positions (RMSD) graph of *Aaeg*OBP1 in APO form (black lines) and complexed with DEET (red), ZINC71773878 (green), and ZINC17917305 (blue) plotted in the XMGrace program. (B) A root‐mean‐square fluctuation (RMSF) graph of *Aaeg*OBP1 in APO form (black lines) and complexed with DEET (red lines), ZINC71773878 (green lines), and ZINC17917305 (blue lines) plotted in the XMGrace program.

This way, 40 ns of MD was applied in order to prioritize the best compound capable of stabilizing the *Aaeg*OBP1 complex in comparison with the APO form and a reference ligand (DEET) complex on trajectory through RMSD of atomic positions (RMSD) by the RMS function implemented on GROMACS (Figure 1A).

The results indicate that all complexes reached structural stability after 20 ns of simulation, exhibiting minimal conformational fluctuations: APO form (RMSD = 3.39 Å; *σ* = 0.48 Å), AaegOBP1–DEET (RMSD = 3.25 Å; *σ* = 0.33 Å), AaegOBP1–ZINC71773878 (RMSD = 3.24 Å; *σ* = 0.70 Å), and AaegOBP1–ZINC17917305 (RMSD = 3.20 Å; *σ* = 0.74 Å). Compared to the APO form, the ligand‐bound complexes exhibited slightly lower RMSD values, likely due to stabilizing intermolecular interactions between the ligands and the AaegOBP1 binding site, which help constrain atomic motion. Although RMSD analysis is considered a global metric of complex stability analysis and, thus, does not allow for assessing the compound's contribution to specific regions (e.g., *Aaeg*OBP1 active site) to system stability [29]. Therefore, to better understand residue‐level dynamics, we further analyzed the behavior of individual *Aaeg*OBP1 residues in both the APO form and in complex with the ligands (Figure 1B).

The root‐mean‐square fluctuation (RMSF) analysis indicates that the atomic fluctuations of key residues in the *Aaeg*OBP1 active site (Phe59, Leu76, Trp114, Tyr112, and Phe123) were reduced in the presence of ligands compared to the APO form. In the unbound state, these residues showed an average RMSF of 1.76 Å (*σ* = 0.46 Å), which remained similar in the presence of DEET (RMSF = 1.76 Å; *σ* = 0.41 Å) but decreased with ZINC71773878 (RMSF = 1.56 Å; *σ* = 0.57 Å) and ZINC17917305 (RMSF = 1.45 Å; *σ* = 0.64 Å). These findings, supported by both RMSD and RMSF analyses, suggest that all ligand‐bound complexes maintained structural stability, with only minor fluctuations in the protein backbone.

RMSD and RMSF are structural metrics that do not account for the energetic contributions of ligands to the overall stability of the system. Therefore, to assess the ability of DEET, ZINC71773878, and ZINC17917305 to stabilize *Aaeg*OBP1, the *g_mmpbsa* module was applied to calculate binding free energies during the production phase of the simulation (20–40 ns), allowing for a more comprehensive evaluation of ligand–protein interactions (Table 2).

**TABLE 2:** Binding free energy of the complexes calculated by *g_mmpbsa*.

Compound	*E*_vdW_	*E*_elec_	*E*_MM_	*G*_polar_	*G*_apolar_	∆*G*
**DEET**	−4.32	21.39	17.07	−42.10	−0.81	−24.13
**ZINC71773878**	−27.96	−5.18	−33.14	22.09	−3.04	−58.29
**ZINC17917305**	−23.47	−3.20	−26.67	13.71	−2.26	−42.64

*Note*: All values are given in kcal/mol.

Abbreviations: ∆*G* = free energy; *E*_elec_ = electrostatic energy; *E*_MM_ = potential energy; *E*_vdW_ = van der Waals energy; *G*_apolar_ = nonpolar solvation energy; *G*_polar_ = polar solvation energy.

On the basis of the g_mmpbsa analysis, the energetic components favorable to intermolecular interactions indicate that ZINC71773878 (*E*_MM_ = −33.14 kcal/mol) and ZINC17917305 (*E*_MM_ = −26.67 kcal/mol) exhibit a stronger binding affinity to *Aaeg*OBP1, as evidenced by their lower molecular mechanics (MM) potential energy (*E*_MM_) values when compared to DEET (*E*_MM_ = 17.07 kcal/mol). These results suggest that both compounds contribute more effectively to the stabilization of the protein–ligand complex.

Although *E*_MM_ values described are favorable to intermolecular interactions, the contribution of desolvation of polar (*G*_polar_) and nonpolar (*G*_apolar_) groups is unfavorable for complex stabilization and, thus, can penalize the binding free energy values [28, 30]. This way, ZINC71773878 (*G*_polar_ = 22.09 kcal/mol) and ZINC17917305 (*G*_polar_ = 13.71 kcal/mol) had higher polar solvation energy values than DEET (*G*_polar_ = −42.10 kcal/mol). However, the nonpolar contributions (*G*_apolar_) were similar for all compounds.

According to *E*_MM_ values presented by the evaluated compounds, the *G*_polar_ penalties were not able to affect the binding free energy and, thus, the complex stabilization. This way, the binding free energy data suggest that ZINC71773878 (∆*G* = −58.29 kcal/mol) and ZINC17917305 (∆*G* = −42.64 kcal/mol) have better values when compared to DEET (∆*G* = −24.13 kcal/mol) and, thus, are able to stabilize *Aaeg*OBP1 during MD. Additionally, when the binding free energy values obtained through the *g_mmbpsa* module are compared with experimental studies of DEET (∆*G* = −7.5 kcal/mol), it is seen that the values of ZINC71773878 (∆*G* = −58.29 kcal/mol) and ZINC17917305 (∆*G* = −42.64 kcal/mol) are higher than the experimental data [20].

On the basis of the best binding free energy value compared to DEET (∆*G* = −24.13 kcal/mol), ZINC71773878 (∆*G* = −58.29 kcal/mol) was prioritized for repellence assays. Additionally, short‐timescale MD simulations (40 ns) were used as an integrated step within the hierarchical virtual screening strategy to evaluate the initial stability of the protein–ligand complexes. This approach allowed us to refine compound selection by identifying candidates capable of maintaining stable interactions with the target under dynamic conditions, thereby supporting the prioritization of ZINC71773878 for biological evaluation.

### 3.1 Binding Mode of Compounds With AaegOBP1 Affinity

**FIGURE 2:**
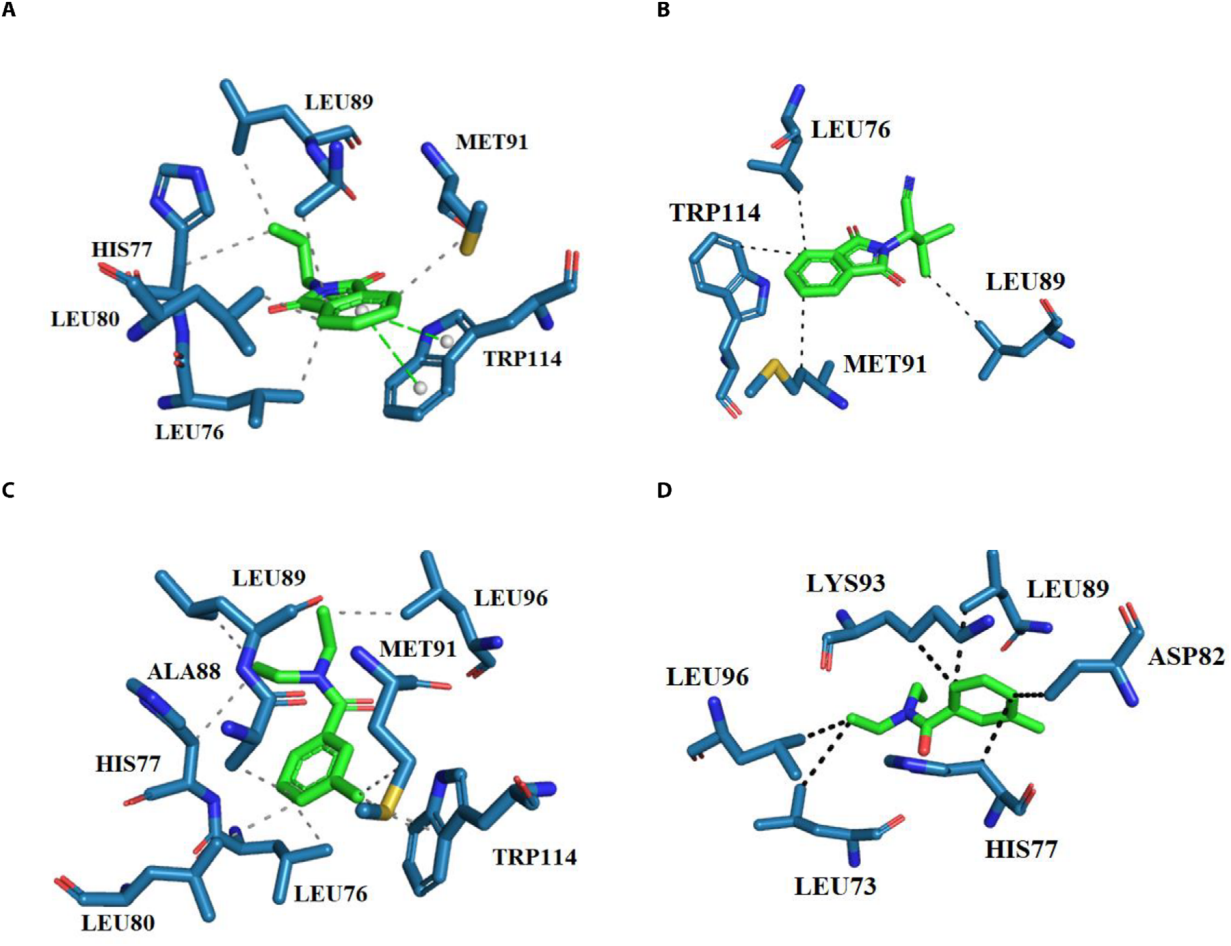
Interaction representation of ZINC71773878 on molecular docking (A) and molecular dynamics (B), and DEET (crystallographic ligand) on molecular docking (C) and molecular dynamics (D) in *Aaeg*OBP1 active site generated by PLIP online server.

In order to show the interaction mode of ZINC71773878 with *Aaeg*OBP1, 2D complexes were generated (Figure 2).

ZINC71773878 compound (Figure 2A; ChemPLP = 56.59) performed two π‐stacking interactions with Trp114 and hydrophobic interactions with residues His77, Leu76, Leu80, Leu89, and Met91 in docking simulation. In comparison with the interactions performed by DEET (crystallographic ligand; Figure 2C), it shows only hydrophobic bonds with Leu76, Phe59, Phe123, Trp114, and Tyr122.

In MD simulations, the most representative cluster of ZINC71773878 (Figure 2B; ∆*G* = −58.29 kcal/mol) performed only hydrophobic interactions with residues Leu76, Leu89, Met91, and Trp114. However, DEET (Figure 2D; ∆*G* = −24.13 kcal/mol) performs hydrophobic interactions with residues Leu96, Leu73, His77, Asp82, Leu89, and Lys93.

The interaction map analysis of the best‐ranked compounds shows the same interaction profile between docking and MD simulation of each compound. In addition, the presence of *Aaeg*OBP1 active site residues suggests the same interaction mechanism between ZINC71773878 and DEET in front of *Aaeg*OBP1, such as Leu89, Leu76, and Trp114 residues. These residues are related as important in complex stabilization with *Aaeg*OBP1 [4, 9, 31].

### 3.2 Repellency Assays

According to the Centers for Disease Control and Prevention (CDC), repellents are compounds applied to skin and surfaces capable of discouraging the mosquito behavior of approaching and feeding on blood; thus, their use reduces the risk of transmission of numerous arboviruses and immunologic reactions resulting from *A. aegypti* bites.

Although repellents are an important ally in reducing cases of arboviruses, the Brazilian sanitary laws require proof of safety and efficacy through biological assays as an alternative to clinical trials [27]. This way, the employment of repellency assays, through the exposure of different dosages of a test compound to human skin, can be useful to verify safety and efficacy, aiming to prioritize a compound for regulatory submission registration as a commercial product.

In addition, despite the integration of in silico approaches guaranteeing success rates higher than random assays and the employment of a validated in silico methodology to prioritize compounds with repellent activity [27], these data are not sufficient to guarantee the affinity of ZINC71773878 in front of *Aaeg*OBP1 and, thus, the modulation of mosquito behavior.

**FIGURE 3:**
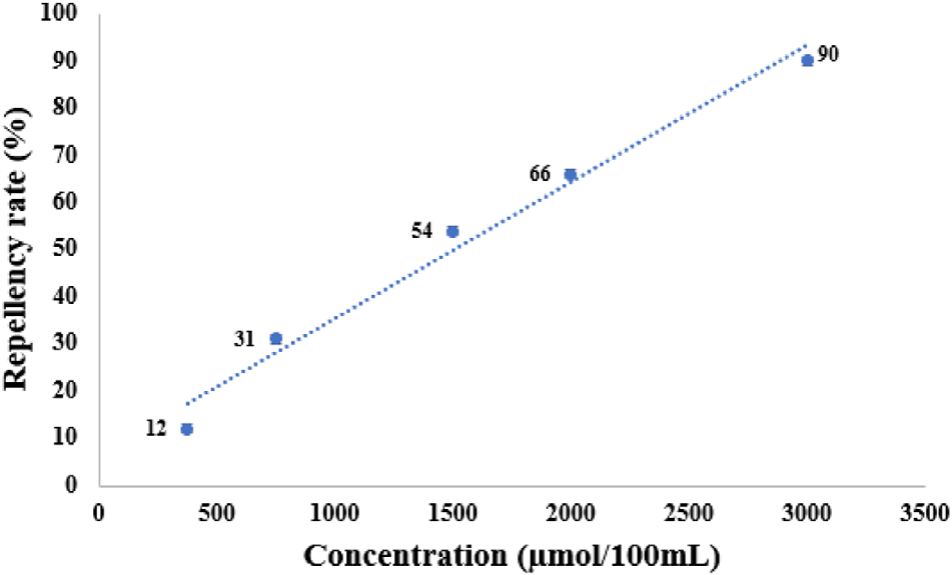
Percentage of mosquitoes repelled by different concentrations of ZINC71773878.

Thus, repellency assays were conducted with five different dosages between the lowest (180 µmol/100 µL) and highest (3000 µmol/100 µL) concentrations capable of modulating the mosquito behavior [9], aiming to visualize a concentration‐dependent response of mosquito exposure to ZINC71773878 (Figure 3).

The results suggest that ZINC71773878 has a linear repellency response with the concentration increase (*R* ^2^ = 0.9818), which may confirm the ability of the compound to bind to *Aaeg*OBP1. Additionally, ZINC71773878 (repellency = 90%) has a higher repellency rate than DEET (repellency = 78%) when compared at a fixed concentration of 3000 µmol/100 µmol. Thus, it suggests that the test compound has a higher repellency activity in comparison with a commercial repellent used as the positive control.

In comparison with several studies, our study was able to demonstrate a repellent activity of ZINC71773878 at lower concentrations than other compounds reported in literature, even when compared with DEET, a standard commercial repellent [4]. In addition, the employment of isolated compounds in repellency assays has a higher potential to identify compounds with better repellency activity than when compared with essential oil assays [32, 33].

Another approach employed to evaluate the dose–response curve of repellency assays is determining the concentrations capable of generating a repellent effect in 50% and 99% of the mosquito population. This approach has been used in several studies in order to define dosages capable of generating a repellent behavior in *A. aegypti* [4, 32, 33, 34]. This way, probit analysis was employed to measure the effective dosage to generate a repellent effect in 50 (ED_50_) and 99% (ED_99_) of mosquitoes.

According to probit analysis (*p* = 0.005; *r* ^2^ = 0.955), ZINC71773878 has ED_50_ = 1210.34 µmol/100 µL and ED_99_ = 10 382.81 µmol/100 µL to generate repellence behavior in 50% and 99% of exposed mosquitoes, respectively. These values show a linear tendency of repellency effects with the dosage increase exposed to *A. aegypti*.

Despite the biological phenomenon observed and the repellency activity of ZINC71773878 being proven, the repellency assays only provide a behavioral response of the mosquito, and it is not possible to understand anatomically how the mechanism of compound presentation to *Aaeg*OBP1 and release of this compound near to the olfactory neuron leads to repellency behavior occurring. Additionally, the lack of information on pH variation influence during the process of compound presentation to *Aaeg*OBP1 (pH = 8.0) and release near the mosquito sensory neuron (pH = 4.0) can be elucidated through MM approaches that use pH as a constant during the simulation time [35, 36].

### 3.3 Constant pH MD

**FIGURE 4:**
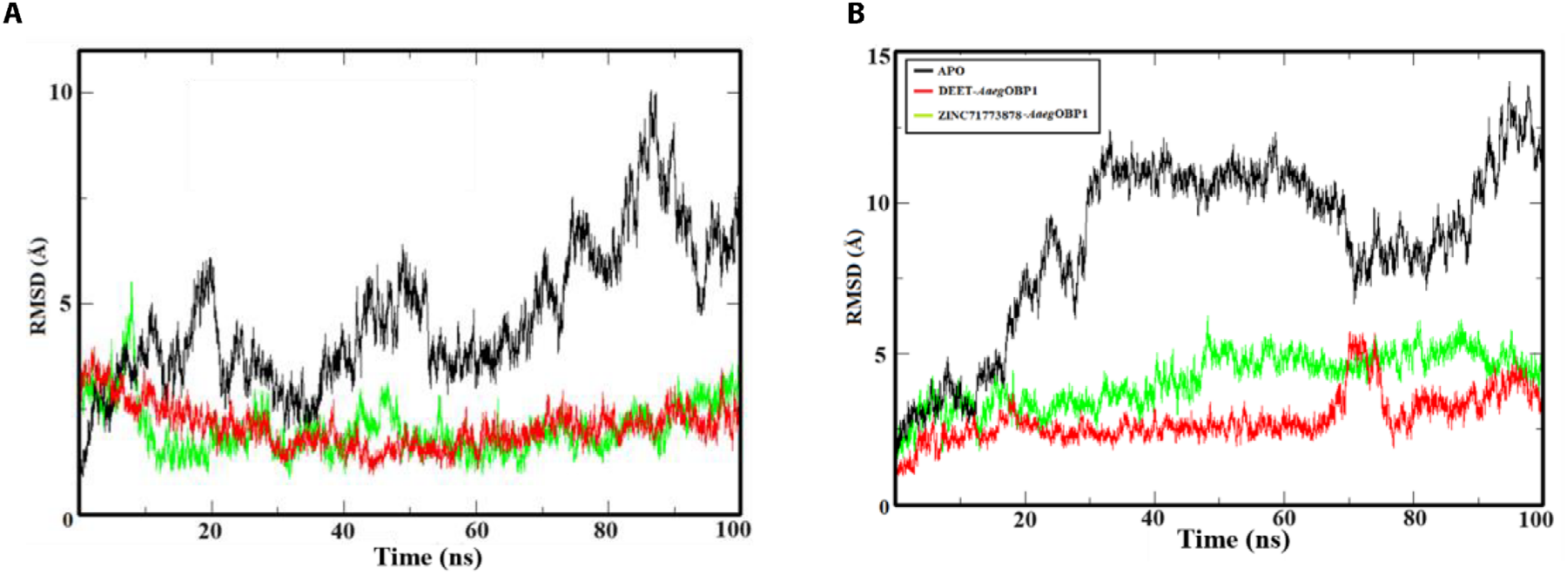
Root‐mean‐square deviation of atomic positions (RMSD) graph of *Aaeg*OBP1 with DEET (red lines) and ZINC71773878 (green lines) compounds in comparison with APO form (black lines) in pH = 8.0 (A) and pH = 4.0 (B) plotted in the XMGrace program.

In order to evaluate the structural stability of *Aaeg*OBP1 complexed with DEET and ZINC71773878 in comparison with APO, we performed CpHMD simulations at different pHs (pH = 8.0 and 4.0). The RMS function implemented on the VMD program was employed to calculate the RMSD of atomic positions of the protein and quantify structural modifications along the trajectories generated on the NAMD module [37] (Figure 4).

The results suggest that at the pH of odor presentation (pH = 8.0; Figure 4A), *Aaeg*OBP1‐DEET (RMSD = 2.072 Å; *σ* = 0.50 Å) and *Aaeg*OBP1‐ZINC71773878 (RMSD = 2.032 Å; *σ* = 0.61 Å) complexes are stable after 10 ns. In comparison with the APO form (RMSD = 4.667 Å; *σ* = 0.47 Å), the complexes had a lower RMSD value due to the presence of intermolecular interactions that establish the compounds in the *Aaeg*OBP1 binding site, which directly affects the variation of atomic positions.

For the odor release pH close to mosquito sensory neurons (pH = 4.0; Figure 4B), *Aaeg*OBP1–DEET (RMSD = 2.83 Å; *σ* = 0.78 Å) and *Aaeg*OBP1–ZINC71773878 (RMSD = 4.151 Å; *σ* = 0.89 Å) complexes also reached stability after 10 ns. The APO form (RMSD = 8.808 Å; *σ* = 2.84 Å) presented a greater atomic perturbation when compared with the RMSD value obtained at pH = 8.0, which shows an unfavorable environment for maintaining intermolecular bonds between the ligands and the macromolecule due to the influence of different pH on the ionizable residues.

When the *Aaeg*OBP1–ZINC71773878 complex stability (Figure 4) is compared at different pHs, a greater atomic variation can be observed during the MD trajectory at pH = 4.0 (RMSD = 4.151 Å; *σ* = 0.89 Å) when compared at pH = 8 (RMSD = 2.032 Å; *σ* = 0.61 Å). The difference in the atomic variation of the complexes can be explained by the influence of the pH in front of the ionizable residues of *Aaeg*OBP1.

The sensillar lymph of the mosquito antenna, at pH = 8.0, offers favorable biological conditions for the formation of a stable *Aaeg*OBP1–ZINC71773878 complex and transport of the odor to the mosquito sensory neuron [9, 38]. However, at pH = 4.0, the sensillar lymph does not offer favorable conditions for the stability of the bonds that occur with ZINC71773878, due to the ionization of the residues present in the *Aaeg*OBP1 binding site. Additionally, the residue ionization of *Aaeg*OBP1 was observed at pH = 8.0 and pH = 4.0 in the simulation of *Aaeg*OBP1–ZINC71773878 complex (Table S2).

The results suggest that at pH 8, there is a greater predominance of non‐protonated residues in the *Aaeg*OBP1 sequence, such as Glu74, His77, and His90, which present 00.00% of ionization during the CpHMD trajectories. Thus, it contributes to maintaining the stability of the *Aaeg*OBP1–DEET and *Aaeg*OBP1–ZINC71773878 complexes. At pH 4, it is observed that these residues are protonated during most of the CpHMD trajectory. Thus, the residues are susceptible to destabilizing interactions, which makes it difficult to maintain the integrity of *Aaeg*OBP1–DEET and *Aaeg*OBP1–ZINC71773878 complexes. In addition, the Lys93 residue is protonated independently of pH.

When the residue ionization is compared with the p*K*a values of each residue, it is observed that the protonation states of Glu 74 A, Lys93 A, and Lys93 B are convergent with the protonation percentage provided by CpHMD at pH 8.0 and 4.0. Additionally, the same convergence is observed for Glu74 B, His77 A, His77 B, His90 A, and His90 B at pH 8.0.

At pH 4.0, CpHMD indicates that His77 A, His77 B, His90 A, and His90 B are protonated during the trajectories, whereas the theoretical p*K*a indicates that these residues should be deprotonated. As CpHMD is a simulation strategy that allows the ionization state of each titratable residue to vary during the simulation, the presence of protons in these residues is an indication of pH influence on the odor release by *Aaeg*OBP1. Thus, it can be suggested that the change in pH and, thus, protonation of the residues can result in subtle conformational changes in *Aaeg*OBP1 and interfere with the stability of the chemical bonds between the odors and the *Aaeg*OBP1 binding site, as shown also by its increase in RMSD (Figure 4).

On the basis of the data provided, CpHMD can elucidate a pH‐dependent mechanism of odor release by *Aaeg*OBP1 at pH 4.0. This mechanism is not visualized in other types of molecular modeling analyses, such as docking or traditional MD.

Several studies indicate that the pH to which proteins of the OPB family are exposed is very relevant for the binding‐release mechanism of odors through the occurrence of conformational changes, such as *A. gambiae* (*Agam*OBP1), *A. aegypti* (*Aaeg*OBP1), and *C. quinquefasciatus* (*Cqui*OBP1) [39]. In these organisms, at pH 4.0, there is a reduction in the affinity of the ligand–OBP complex due to the ionization of titratable *N*‐terminal residues that expose the hydrophobic cleft to the acidic sensillar lymph, as highlighted by the ionization changes of Glu74, His77, and His90 in the present study [11].

Despite the stability information, RMSD and residue ionization evaluation are strategies for evaluating the protein only and do not provide information about the energetic contribution of compounds to system stabilization. One approach that can be employed to evaluate the process of odor presentation and release by *Aaeg*OBP1 is through the sum of non‐binding energy contributions (e.g., VdW and Coulomb interactions) [40]. Thus, the compound‐*Aaeg*OPB1 dissociation process can occur when there are variations in the total energy value of the system. Thus, the energy contributions of *Aaeg*OBP1–ZINC71773878 complex were analyzed to understand the effect of different pHs (pH = 4.0 and pH = 8.0).

### 3.4 Dissociation Energy Contributions

The energetic contributions of *Aaeg*OBP1–ZINC71773878 complex were investigated using the PLUMED code implemented in NAMD program, under the NPT ensemble, to represent in a reduced manner the dissociation free energy at different pHs (Table 3).

**TABLE 3:** Energy contributions of the *Aaeg*OBP1–ZINC71773878 complex under the effect of different pH during constant pH molecular dynamics (CpHMD).

pH	Electrostatic energy	Van der Walls Interaction	Total energy
pH = 4.0	−16.54	−29.50	−46.04
pH = 8.0	−3.84	−34.13	−37.98

*Note*: All values are given in kcal/mol.

At pH 4.0, the electrostatic energy is more negative (−16.54 kcal/mol), indicating stronger electrostatic interactions between ZINC71773878 and *Aaeg*OBP1. At pH 8.0, the electrostatic energy becomes less negative (−3.84 kcal/mol), suggesting a reduction in the electrostatic contribution to the stability of the complex. This effect may occur due to variations in the protonation state of ionizable residues at different pHs.

When VdW interactions are evaluated, at pH 8.0, the VdW energy is more negative (−34.13 kcal/mol), indicating stronger interactions between ZINC71773878 and *Aaeg*OBP1. At pH 4.0, the VdW energy is less negative (−29.50 kcal/mol), suggesting a weaker interaction.

Overall, these findings suggest that at pH 4.0, the complex is primarily stabilized by electrostatic interactions, as they are stronger, whereas VdW interactions are weaker. Conversely, at pH 8.0, electrostatic interactions are reduced, whereas VdW interactions intensify, indicating that the stability of the complex is now predominantly determined by hydrophobic forces.

Additionally, because VdW interactions are stronger at pH 8.0, the dissociation of the complex may be more difficult at this pH, suggesting greater stability. At pH 4.0, the less negative VdW energy may result from differences in the protonation state of ionizable residues in *Aaeg*OBP1, which may correlate with the RMSD, RMSF, and percentage of ionization data presented previously.

According to stability and energy analysis provided by CpHMD trajectories, ZINC71773878 is able to bind and stabilize *Aaeg*OBP1 and, thus, corroborate with repellency assays [5, 10, 41]. In order to elucidate the binding mode of the compound with repellent activity and the presence of hydrogen bonds that help to establish the complex, the permanency time of H_bond and the interaction map of *Aaeg*OBP1–ZINC71773878 complex were evaluated.

### 3.5 Permanency Time of H‐Bond and Interaction Map Analysis

The permanency time of hydrogen bonds (H‐bonds) formed between ZINC71773878 and *Aaeg*OPB1 was performed in order to elucidate the residues that stabilized the compound during CpHMD simulation and also to evaluate the changes in this pattern when the pH of the medium changes (Table S3).

According to data analysis, at pH = 8.0, only the residue Phe123 (36.33%) interacts with ZINC71773878 for more than 10% of the simulation time of the productive phase. This interaction occurs mainly with the ketonic group of the imine present in ZINC71773878 (acceptor), and, thus, it can be inferred that Phe123 is responsible for stabilizing ZINC71773878 in the *Aaeg*OPB1 binding site during the CpHMD simulation at pH = 8. Additionally, at pH = 8.0, Phe123 is deprotonated and, thus, has a favorable protonation state for ZINC71773878 interaction.

At pH = 4.0, only the His77 (14.27%) residue interacts with ZINC71773878 for more than 10% of simulation time, and there are no significant occurrences of hydrogen interactions with the residues of the *Aaeg*OBP1 binding site. Additionally, the His77 residue is not involved in the binding site of *Aaeg*OPB1 [9].

**FIGURE 5:**
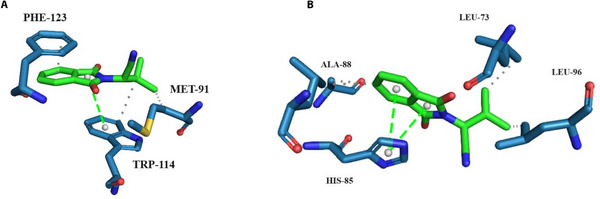
Interaction maps of *Aaeg*OBP1–ZINC71773878 complex of the representative cluster of CpHMD at pH = 8.0 (A) and pH = 4.0 (B) generated by the online software PLIP. The gray dashed lines correspond to hydrophobic bonds. The green dashed lines represent π‐stacking interactions.

In this perspective, the graphical representation of the interaction profiles presented by *Aaeg*OBP1–ZINC71773878 complex at different pH levels is important for the design of compounds with repellent activity and for understanding the pH‐dependent mechanism of ligand presentation to NSO. For this purpose, the binding mode of the *Aaeg*OBP1–ZINC71773878 complex at each pH was proposed through the average structure of the most populated cluster using a cutoff of 0.15 nm (Figure 5).

According to the interaction map of average structure during the productive phase of CpHMD at pH = 8.0, ZINC71773878 has hydrophobic interactions with Phe123 and Met91 residues and a π‐stacking interaction with Trp114 residue (Figure 5A). This pattern of interactions is repeated when compared with the interaction map of molecular docking and traditional MD (Figure 2A,B).

The interaction map of average structure during the productive phase of CpHMD at pH = 4.0 (Figure 5B) shows that ZINC71773878 performs hydrophobic interactions with Ala‐88, Leu73, and Leu96 residues and a π‐stacking interaction with His‐85 residue. When the interaction maps of the *Aaeg*OBP1–ZINC71773878 complex are compared, it is clear that the residues involved in the stabilization of ZINC71773878 in the *Aaeg*OPB1 binding site are not evident due to the ionization effects at pH = 4.0. Additionally, the ionization of these residues may be related to the pH‐dependent mechanism of ligand release near the NSO.

On the basis of the CpHMD strategies employed, it is suggested that the pH variation of the sensillar lymph of *A. aegypti* influences the protonation of *Aaeg*OBP1 residues and, thus, the presence of higher dissociation energy contributions at pH = 4.0 (dissociation energy = −46.04 kcal/mol) than when compared at pH = 8.0 (dissociation energy = −37.98 kcal/mol). Additionally, the influence of different pHs can be observed in the hydrogen bond residence time and profile of the intermolecular interaction maps, where at pH 8.0, the presence of *Aaeg*OBP1 binding site residues is evident, whereas at pH 4.0, these residues are not evident.

This way, the repellency activity of ZINC71773878 in front of *Aaeg*OBP1 was proved by constant pH simulations at two different pHs. It is worth remembering that the OBP family is present in several vectors with medical importance, such as *Aedes albopictus*, *A. gambiae*, and *C. quinquefasciatus*, and, thus, ZINC71773878 can have biological activity in front of these other vectors.

In addition, ZINC71773878 showed efficacy against *A. aegypti* and should be prioritized in subsequent trials (such as protection time and formulation assays) aiming for the regulatory registration of a new product with repellent activity that is more safer and more efficient in comparison with DEET.

### 3.6 Pharmacotechnical Prediction of Novel Repellents

The development of novel products with repellent activity has its roots deeply linked to the planning stages of stable formulations capable of maintaining the safety, efficacy, and quality criteria recommended by regulatory agencies. The repellency assays showed that ZINC71773878 in controlled doses in a hydroalcoholic solution was able to demonstrate a repellent effect in front of *A. aegypti*. Thus, ZINC71773878 is a promising compound for the design of new formulations with repellent activity. This way, the scope of a formulation for ZINC71773878 was predicted, as well as the possible behavior of the formulation at different temperatures.

The design of repellents in liposomal formulations is of great interest and has been extensively investigated in the pharmaceutical sector as safe and effective transport systems for volatile compounds. In particular, among the various pharmaceutical applications of liposomes, the use of cyclodextrins has a special interest in the formulation of topical drugs, due to their effectiveness in encapsulating compounds [42]. ZINC71773878 was evaluated for its ability to form a stable complex with different types of cyclodextrins through the FormulationAI online server [43] (Table S4).

According to the energy values, ZINC71773878 has been shown to form stable complexes with the various types of cyclodextrins available. Among them, it is worth highlighting Hp‐β‐CD, which is used in the development of topical formulations (such as sunscreens and repellents) because it considerably reduces the systemic absorption of the active components of the formulations [44]. Thus, the use of Hp‐β‐CD can reduce the possibility of dermal irritation, since in its presence, only a part of the compound is free, due to the equilibrium between it and its bound form.

In addition, it was simulated that phospholipid system ZINC71773878 best adapts to make a stable liposome complex. In this perspective, ZINC71773878 demonstrated a better stability profile in a liposomal formulation composed of phosphatidylglycerol (20%), phosphatidylcholine (46.7%), and Tween 80 (33.3%).

The proposed liposomal system has a dispersion index of 0.17, a zeta potential of −25.51 mV, and a retention percentage of 60.66% in the skin. Thus, it is suggested that the concentrations of the proposed continuums are capable of retaining ZINC71773878 in the liposomal system in a stable complex.

Another relevant quality criterion in the design of novel formulations is the evaluation of storage stability. Traditionally, the stability of a repellent formulation can be evaluated through its organoleptic characteristics, that is, through sight, smell, and touch. These senses allow an analysis of the chemical composition of the sample, thus preventing the instability of the formulations from spreading to consumers. On the other hand, the employment of computational approaches capable of predicting the behavior of chemical compounds in relation to the main polymers present in cosmetic formulations and at different temperatures has been used as a preliminary and rational evaluation strategy for the development of new products [45]. In this perspective, the behavior of ZINC71773878 was evaluated at storage temperatures of 25°C, 37°C, 40°C, 45°C, and 50°C to simulate its stability over a period of 90 and 180 days (Table S5).

According to results, ZINC71773878 demonstrated chemical and physical stability in the presence of polymers PEG8000 and PEG2000 at room temperature (25°C) and at the temperatures used for stability assays for cosmetic products (37°C, 40°C, 45°C, and 50°C) for a period of 90 days [46]. For the period of 180 days, the stability of ZINC71773878 with PEG8000 and PEG2000 was maintained.

This way, the simulation of the proposed stability tests is an alternative for optimizing resources and time spent on pre‐formulation assays and can predict the behavior of ZINC71773878 based on the specific climatic characteristics of subtropical regions, where the highest number of cases of arboviruses are commonly found.

Thus, in silico simulations of pre‐formulations will ensure greater elucidation during the prioritization of the best constituents for the development of new repellent products, as well as a prediction of the physical–chemical behavior of ZINC71773878 in temperature variation exposure. It is worth noting that the results described in this chapter are in accordance with the ICH Q1A(R2) Guide for Stability Testing of New Drug Substances and Products and the Anvisa Cosmetic Product Stability Guide [46].

## 4 Conclusions

The employment of a previously validated in silico methodology was able to prioritize non‐toxic compounds with stereo‐electronic requirements and binding affinity to AaegOBP1. Additionally, the application of physicochemical and skin sensitization prediction was able to prioritize compounds with the same efficacy and safety requirements as commercial repellents.

Virtual screening by pharmacophore model and molecular docking allowed the prioritization of 1271 compounds from Sigma‐Aldrich (https://www.sigmaaldrich.com) and the Biogenic dataset of metabolites and NPs from the ZINC15 platform. Of these, five compounds did not have penalties in the physicochemical and skin sensitization prediction. The traditional MD generated from the GROMACS package provided an analysis of the stability behavior and free energy binding of ZINC71773878 and ZINC17917305 through RMSD, RMSF values, and free energy binding estimation. By comparing the poses obtained in molecular docking and the representative MD cluster, it was observed that DEET, ZINC00131924, and ZINC00170981 remained in the AaegOBP1 binding site throughout the simulation time. According to the best binding free energy value, ZINC71773878 (∆*G* = −58.29 kcal/mol) was prioritized for repellency assays.

The methodology employed to perform the repellency assays was able to evaluate ZINC71773878 to modulate the behavior of *A. aegypti* at different concentrations. The repellency data suggest that ZINC71773878 has a linear repellency response with increasing concentration, which may confirm the capacity of these molecules to bind to AaegOBP1.

The CpHMD approach was able to elucidate the energetic contributions and the effects of different pHs to AaegOBP1–ZINC71773878 complex dissociation at pH = 4.0. In addition, the permanency time of hydrogen bonds and interaction map analysis showed different interaction patterns at pH = 8.0 and pH = 4.0.

This way, the repellency activity of ZINC71773878 in front of AaegOBP1 was probed, and this compound must be prioritized for further trials aiming at the development of a new product with repellent activity that is more safer and more efficient than the available commercial repellents in Brazil.

It is noteworthy that ZINC71773878 can also have repellence activity in *A. albopictus*, *A. gambiae*, and *C. quinquefasciatus*, because these vectors share the same OPB family in the transmission of chemical signals to NSO for behavior modulation.

## 5 Experimental Section

### 5.1 Dataset

A set of 24 molecules with *A. gambiae* odorant‐binding protein 1 (AgamOBP1) affinity available in the literature with a Ki value [6] was catalogued into training and test sets. Marvin Sketch 16.9.5 software [47] was used to draw the 2D structures and select the most reliable tautomers in presentation pH (8.0) [5, 9]. Subsequently, the CONCORD module, implemented on SYBYL‐X 2.0 package [23], was used to convert the structures to 3D format. All structures had their energy minimized through the conjugate gradient (CG) method using a convergence criterion of 0.001 kcal/mol and Tripos force field (with dielectric constant *ε* = 80.0 and maximum number of iterations = 50 000) [48]. Partial atomic charges were calculated using the Gasteiger–Hückel method [49], available on the SYBYL platform [23].

In order to select the best training set of compounds able to generate a reliable pharmacophore model, a chemical similarity study was carried out. The compounds were selected according to chemical diversity estimated by the ChemGPS‐NP web server [50]. The first three principal components (PC1, PC2, and PC3) were used to build the dendrogram using the centroid method and Tanimoto Index (similarity > 65%) as measurement parameters by MINITAB software [51] (Figure S5).

The most potent compounds of each cluster of dendrogram were selected for the training set (Table S6). However, the remaining compounds were selected for the test set.

### 5.2 Pharmacophore Generation

The conformers were obtained using Genetic Algorithm (GA) method by Linear Algorithm for Hypermolecular Alignment of Data Sets (GALAHAD), implemented on the SYBYL platform [23]. The pharmacophore features were generated by the flexible superimposition of training set compounds to create hypermolecular alignments. The GA employed in this step starts with 40 conformations (population size) of each compound that evolve through a maximum of 50 generations through genetic operators adjusted (mutation rate—angle: 1.0, Conf: 1.0; mutation drop—angle: 1.0, Conf: 1.0; and crossover rate—angle: 1.0, Conf: 1.0), such as implemented in GALAHAD module from SYBYL‐X 2.0.

### 5.3 Pharmacophore Model Evaluation

Pharmacophore models with high strain energy (>100 kcal/mol) were discarded. Then, the remaining models were evaluated by Pareto score. The discriminatory power to recognize actives and decoys was used to evaluate pharmacophore models statistically similar (Pareto = 0), and thus, DUD‐E server [52] was used to generate decoys and the SigmaPlot program v. 12.0 [53] to calculate the AUC of each ROC (ROC curve).

#### 5.3.1 Molecular Docking Studies

The 3D structures of AaegOBP1 (PDB: 3K1E) [9] and AgamOBP1 (PDB: 3N7H) [6] were aligned by the Biopolymer module available on SYBYL‐X 2.0 platform using the command “align structures by Homology with MSA” function [23]. The sequential identity and RMSD were calculated between the structures. Pymol 2.2.3 software was used to verify the position of residues involved in the active site of AaegOBP1 and AgamOBP1.

The 3D structures of AaegOBP1 (PDB: 3K1E) and AgamOBP1 (PDB: 3N7H) were aligned by Pymol 2.2.3 software. The crystallographic ligand present in AgamOBP1 was extracted to AaegOBP1, which was used in a molecular docking study.

The AaegOBP1 structure obtained by cross‐docking employed in the molecular docking study was prepared by the Biopolymer module from the SYBYL‐X 2.0 platform [23]. All ions and water were discarded, and hydrogen atoms were added. Then, the protonation state of residues was manually checked using the H++ server [54] with pH = 8.0 [9].

The conformational search and scoring evaluation were performed by Piecewise Linear Potential (ChemPLP), GoldScore, ChemScore, and Astex Statistical Potential (ASP) scoring functions available on GOLD program 5.4.0 using the default parameters [24]. The ability to generate satisfactory solutions of scoring functions was probed by the RMSD value (RMSD < 2 Å).

#### 5.3.2 Hierarchical Virtual Screening

**FIGURE 6:**
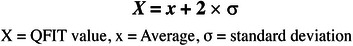
Mathematical equation of the mean plus two times the standard deviation.

The best AgamOBP1 pharmacophore model was used to filter the ZINC database available on the Sigma‐Aldrich platform and Biogenic Database. The molecules with QFIT values above the mean of the QFIT values plus twice the standard deviation (Figure 6) were selected for molecular docking, physical–chemical, and skin sensitization analysis.

X=x+2×σ

#### 5.3.3 Physical–Chemical and Skin Sensitization Virtual Screening

A set of 29 molecules with affinity to AaegOBP1 [9] was submitted to Marvin Sketch 15.4.20 program [47] to calculate the following descriptors: MW, PSA, hydrogen bond acceptors (HBA), HBD, and the partition coefficient (Log *P*). The compounds prioritized on virtual screening were used in this step. Additionally, ADMETlab 3.0 online server [55] was used to predict the skin sensitization of the prioritized compounds.

#### 5.3.4 Molecular Dynamics

GROMACS 4.5.6 version package (University of Groningen, Groningen, Groningen Province, the Netherlands) [56] was used to perform MD simulations of the APO form and complexes, which were obtained from the previous docking step. The topology of each ligand was generated using the ATB 1.0 server and used to build the complexes.

GROMOS96 force field (53a6) [56] was employed for all simulations. The water molecules (extended simple point charge [SPC/E] model) (BERENDSEN et al. 1987) [57] were inserted into a cubic box at a 1.4 nm distance from the protein surface. Some water molecules were replaced by positive ions (Na^+^) to neutralize the system and were randomly distributed inside the box.

A three‐step procedure (5000 steps each) of energy minimization was employed to prepare the system for production MD. First, a steepest‐descent algorithm was applied, restraining harmonically the protein non‐hydrogen atoms to their initial positions, followed by a second steepest descent minimization with all atoms unrestrained. Subsequently, a conjugated gradient algorithm was applied to the entire system for further energy minimization. The bonds involving hydrogen atoms were constrained using LINCS and SETTLE algorithms for protein/ligands and water molecules, respectively. Periodic boundary conditions (PBCs) were applied to Coulomb and VdW interactions.

The long‐range interactions were treated using the particle‐mesh Ewald (PME) electrostatic method. A 1000 ps MD equilibration was performed with the protein non‐hydrogen atoms’ position restrained. In this step, a random Boltzmann distribution was used to generate the initial velocities for each simulation. Then, each simulation of complexes was performed for 40 000 ps [22, 28] at constant temperature (303.15 K) and pressure conditions (1 atm). The average structure was selected by a clustering algorithm method (DAURA; VAN GUNSTEREN; MARK, 1999) implemented in GROMACS 4.5.6, with a cutoff of 0.15 nm during the productive phase.

#### 5.3.5 Trajectory Analysis

RMSD of atomic positions was employed to evaluate the structural stability of the APO form and complexes by the RMS function implemented in GROMACS 4.5.6 [56]. Next, an RMSF was employed to evaluate the residue fluctuation of the APO form and complexes by the RMS function implemented in GROMACS 4.5.6.

#### 5.3.6 Binding Free Energy

The MM Poisson–Boltzmann surface area (MM/PBSA) method implemented in the g_mmpbsa tool was employed to quantify the binding free energy of AaegOBP1 for each compound employed in the MD study in 40 snapshots extracted every 0.5 ns from the production trajectories (20–40 ns). The vacuum potential energy (MSE) was measured by electrostatic (Eelec) and VdW (EvdW) interactions using Coulomb and Lennard–Jones (LJ) potential functions. However, the polar solvation energy (Gpolar) of the complexes was quantified in a lattice box (cfac = 2 and fadd = 20) with 0.150 M NaCl solvent (radiiNa = 0.95 Å; radiiCl = 1.81 Å) and dielectric constant = 80 by Debye–Hückel approximation. The nonpolar solvation energy (Gnonpolar) was calculated using a solvent‐accessible surface area (SASA) model with a predetermined solvent surface tension (gamma = 0.02267 kJ mol^−1^ Å^−2^). The standard output provides the binding free energy value of each complex. The compound with the best free energy value and available for acquisition on Sigma‐Aldrich was prioritized for repellency assays.

#### 5.3.7 Repellency Assays

Repellency assays were performed according to the Klun and Debboun methods [58].

#### 5.3.8 Ethical Aspects

This study was performed in accordance with Resolution 466/12 of the National Health Council [59]. The volunteers were informed about the aims of the research, approaches employed, recording and analysis of data obtained, and the dissemination of results to scientific publications. Anonymity and autonomy were guaranteed to volunteers during the experiments. This work was assessed and approved by the Ethics Committee of the Federal University of Western Bahia (N 3.610.146).

#### 5.3.9 Prototype Construction

The design and construction details of the prototype employed in repellency assays are described in Figure S6.

Each cage had a covered access hole for transferring mosquitoes to the cage and a bottom with a 3 by 4 cm rectangular hole that opened and closed with a sliding door. The concave bottom of the prototype adapted to the curvature of the volunteer's forearm. A separate lower section of the same dimensions served as a skin marking model. The constructed prototype had six constructed cages (Figure 3).

#### 5.3.10 Insects

Female *A. aegypti* mosquitoes from the Henrique Insect Laboratory, located at the Institute of Health Sciences, Federal University of Bahia, were used. The females used (aged 3–7 days) were subjected to a blood fasting for 24 h before the repellency tests. The repellency assays were performed under controlled environmental conditions (temperature = 27°C and humidity = 60%).

#### 5.3.11 Compounds

The compounds employed in the repellency assays, (2*S*)‐2‐(1,3‐dioxo‐1,3‐dihydro‐2*H*‐isoindol‐2‐yl)‐3‐methylbutanenitrile (ZINC71773878) and DEET (commercial repellent), were purchased commercially from Sigma‐Aldrich. The compound ZINC71773878 was diluted using ethanol at concentrations of 3000, 2000, 1500, 750, and 375 µmol/mL to verify the repellency activity of different concentrations of each compound. DEET was employed in a constant concentration (3000 µmol/mL) to validate the repellency methodology during the replicates of the repellency assays.

#### 5.3.12 Repellency Assay Conduction

Repellency assays were performed on three adult human volunteers (two males and one female) with no sensitivity to mosquito bites. During the assay preparation, the test area of volunteers’ skin was washed with unscented soap and rinsed with water, then rinsed with 70% ethanol solution and dried with clean towels. Considering the possibility that several factors may alter mosquito behavior and may affect the results of repellency assays, volunteers were asked not to use fragrances and repellent products for 12 h before and during the assay.

During the assay, 20 adult females of *A. aegypti* aged 3–7 days after emergence were placed inside the first four cages of the prototype constructed (5 females per cage). The test began with the application of 100 µL of ethanol and three solutions of the test compound at different concentrations applied to an area of 12 cm^2^ of skin on the forearm for each solution, between the wrist and elbow of the volunteer, and dried by evaporation for 60 s, according to Figure S7.

The prototype with the females of *A. aegypti* was positioned respectively in the areas where the solutions were applied, and the cages were opened for 120 s to evaluate the rate of blood meals and landings in the exposure area. The influence of the lowest and highest concentrations described as necessary for the repellent effect was investigated [9]. The procedure was repeated again using the two highest concentrations of the compound tested, according to Table S7.

The commercial repellent DEET at 3000 µmol/mL in ethanolic solution was employed as a positive control, whereas ethanol was employed as a negative control. The assays were repeated in triplicate, and the experimental data obtained were analyzed using PROBIT analysis in the SPSS program to measure the effective dosage to generate a repellent effect in 50% (ED_50_) and 99% (ED_99_) of the mosquito population.

In order to understand the observed repellency effects atomically, the tested compound with repellent activity was employed in MD study at constant pH.

### 5.4 Constant pH MD

#### 5.4.1 Ligand Parameterization

3D coordinate of each compound with repellent activity, obtained through the molecular docking step, was submitted to the ATB 1.0 online server for topology generation. The parameters of atomic charge, bond length, and torsional and dihedral angles were obtained using the CHARMM36 force field.

#### 5.4.2 CpHMD Simulation

CpHMD simulations of 100 ns were performed on the namdcph module implemented in the NAMD program [60] using the CHARMM36 force field, at *T* = 298 K and a pressure of 1 atm and at pHs 8.0 and 4.0. The SETTLE algorithm was applied in order to preserve the geometry of the water molecules, and the SHAKE algorithm was applied to fix the length of the covalent bonds. Non‐bonded and short‐range interactions were truncated at 12 Å with a switching distance of 10 Å. Long‐range interactions were treated with the PME method.

#### 5.4.3 Trajectory Analysis

Initially, the structural stability of the APO form and the complexes was investigated by the RMSD using the VMD program. Then, the residue ionization analysis during the trajectory was evaluated, and the average values of total, electrostatic, and VdW energies were calculated, using the namdenergy module of the VMD program, for the complexes in order to observe the stabilization capacity of the molecules against AaegOBP1.

The complexes considered stable were subjected to the study of intermolecular interaction analysis using the online server Protein‐Ligand Interaction Profiler (PLIP) and permanency time of hydrogen bonds (H‐bond) interactions.

#### 5.4.4 Pharmacotechnical Prediction of New Repellents

In order to predict a stable formulation, the best compound prioritized in the steps described above was evaluated for the ability to make a stable complex in the presence of liposomal constituents (binding energy < 0 kJ/mol) employing the Drugs/CDs complexation module implemented on FormulationAI online server [43].

In addition, the properties of liposome module of FormulationAI online server [43, 61] were employed to predict the best liposomal system for the repellent formulation. The best liposomal system was considered with a dispersion index close to 0.0 and a zeta potential close to −30.00 mV [43, 61].

The storage stability of the best compound with repellent activity was simulated with the main polymers used in cosmetic formulations. For this, the solid dispersion stability module of FormulationAI online server [45] was employed to predict the chemical stability at 25°C, 37°C, 40°C, 45°C, and 50°C temperatures and relative humidity = 75% for 90 and 180 days (ICH, 2003; Anvisa, 2020). The formulation was considered stable with a stability percentage > 0.50 (Table S‐8).

## Author Contributions

**Moysés Fagundes de Araujo Neto**: outlined the computational steps, discussed and analyzed the results, wrote the article. **Jânio Rodrigo de Jesus Santos**: carried out the bioassays, discussed and analyzed the results. **Ana Luiza Pontes Silva de Oliveira**: carried out the bioassays. **Antonio Wanderson Vieira Gois**: carried out the bioassays. **Ingrid Bernardes Santana Martins**: carried out the constant pH molecular dynamics, discussed and analyzed the results. **Vitor Barbanti Pereira Leite**: carried out the constant pH molecular dynamics. **Adriano Figueiredo Monte Alegre**: developed the concept of the work. **Jairo Torres Magalhães Junior**: developed the concept of the work. **Franco Henrique Andrade Leite**: developed the concept of the work.

## Conflicts of Interest

The authors declare no conflicts of interest.

## Supporting information




**Supporting File 1**: cbdv70655‐sup‐0001‐SuppMat.pdf

## Data Availability

The data that support the findings of this study are available on request from the corresponding author. The data are not publicly available due to privacy or ethical restrictions.
