# Supplementary material for: Development of a Novel Aedes aegypti Repellent: An Integrated Approach Combining In Silico and Bioassays
Source: Chem Biodivers. 2025 Dec 3;23(1):e01251. doi: 10.1002/cbdv.202501251 (PMC12761363; doi:10.1002/cbdv.202501251)
Supplement: Supplementary file 1 — Supporting File 1: cbdv70655‐sup‐0001‐SuppMat.pdf [file CBDV-23-e01251-s001.pdf]

Supplementary data

Figures

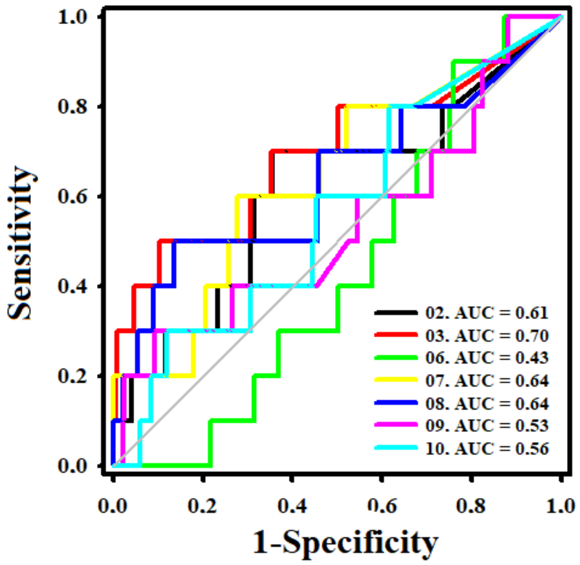

Figure 1. ROC curve of pharmacophore models with low strain energy. The diagonal line represents a model that would not be better than a random selection (AUC < 0.5).

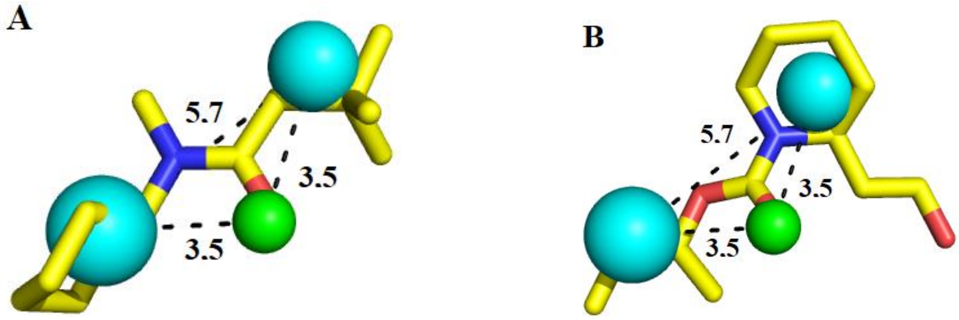

Figure 2. Potent *Agam*OBP1 modulator (A, Ki = 4.18 µM) and commercially available repellent (B, Ki = 0.034 µM), superimposed to pharmacophore model 03. Green spheres represent Hbond acceptor group and cyan spheres are hydrophobic groups. The size of spheres varies according to tolerance radius calculated using GALAHAD. All the distances are measured in Angstroms.

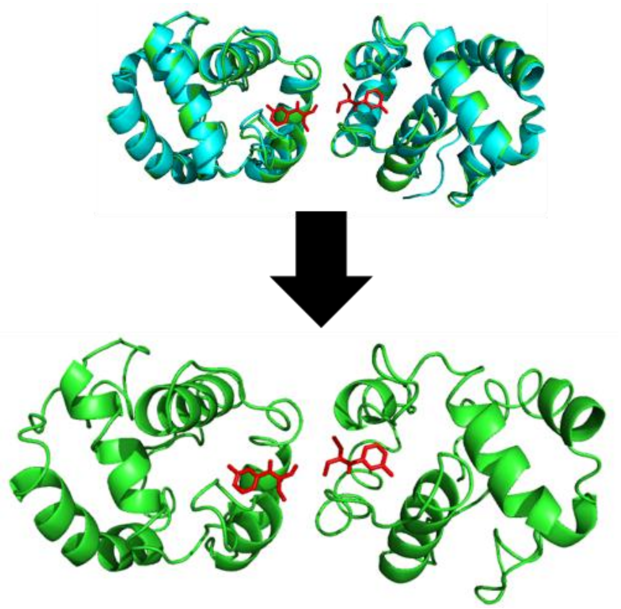

Figure 3. Cross-docking representation using *Aaeg*OBP1 (green) and *Agam*OBP1 (cyan) macromolecules. The chemical structure in red represents the crystallographic ligand (DEET).

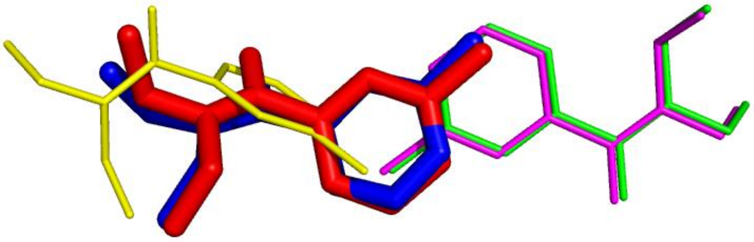

Figure 4. RMSD representation of GOLD fitness functions. Red structure: crystallographic ligand; Blue structure: ChemPLP; Yellow structure: GoldSCORE; Magenta structure: ChemSCORE; Green structure: ASP.

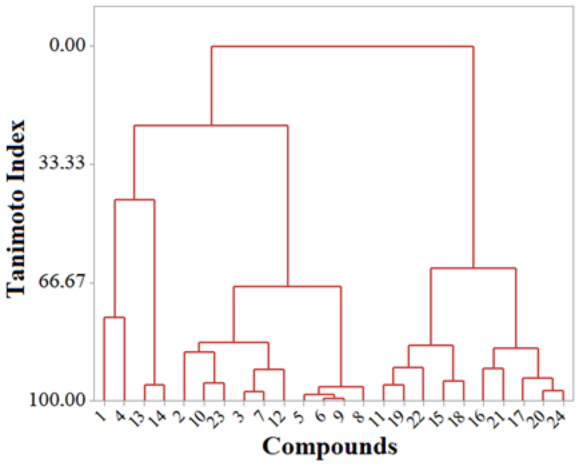

Figure 10. Dendrogram built to AgamOBP1 similarity study.

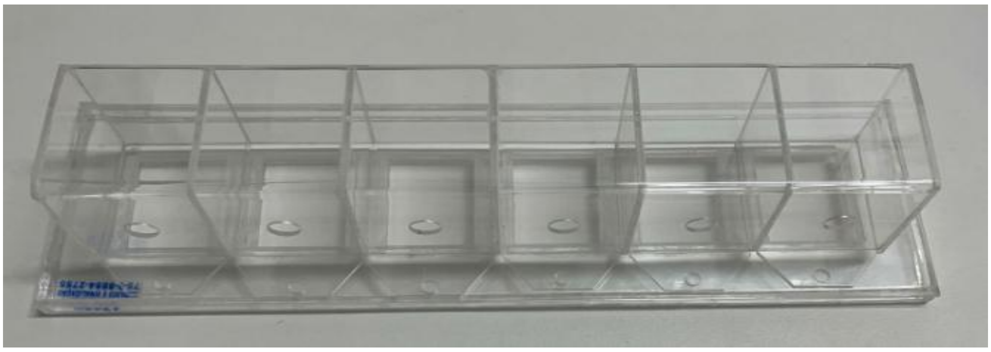

Figure 11. Design of the prototype built to perform the repellency assays adapted from Klun and Debboun[57]. The prototype has 0.3 by 5.6 by 26.2 cm top; 0.3 by 5 by 26.2 cm bottom; 0.3 by 5 by 5 cm partitions (6 partitions); 0.3 by 5 by 26.2 front with six 1 cm diameter holes each; 0.63 by 7 by 30.2 cm base with six 3 by 4 cm doors in the center of each cage.

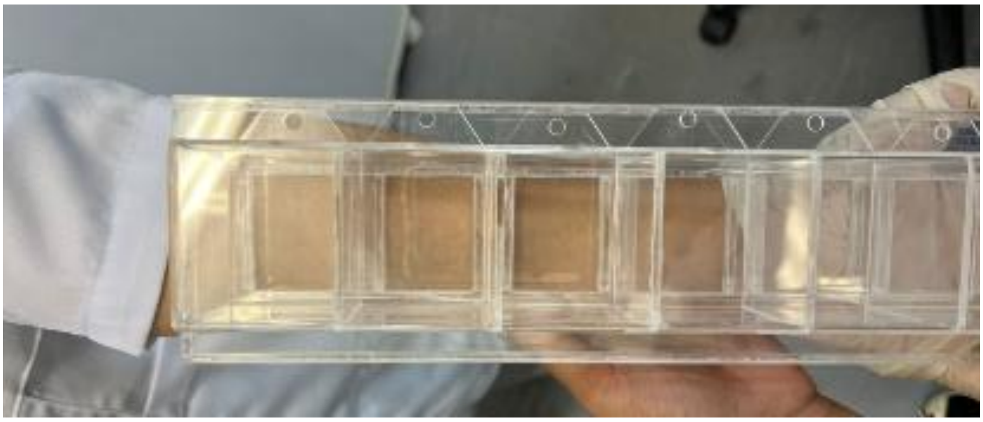

Figure 12. Representation of bioassay test.

Tables

Table 1. Statistical parameters of *Agam*OBP1 pharmacophore models.

Model	Energy[a]	Sterics	H_bond	Mol_qry	Pareto
**01**	415.68	36.90	01.40	00.00	00.00
**02**	16.52	24.70	01.00	00.00	00.00
**03**	14.50	19.20	00.90	00.30	00.00
**04**	13.67	24.20	00.30	00.30	00.00
**05**	415.68	36.90	01.40	00.00	00.00
**06**	10.75	20.30	00.60	00.30	00.00
**07**	16.40	26.00	00.70	00.00	00.00
**08**	112.32	26.50	01.80	00.00	00.00
**09**	10.69	20.60	00.30	00.26	00.00
**10**	13.92	24.60	00.70	00.00	00.00
[a] Kcal/mol					

Table 4. Residue ionization of *Aaeg*OBP1 with DEET and ZINC71773878 compounds in comparison with APO form in pH = 8.0 and pH = 4.0. All values are given in %.

pH	APO	DEET	ZINC71773878	pKa
Glu74 A				4.14
4.0	35.30	88.90	98.60	
8.0	00.00	00.00	00.00	
Glu74 B				3.76
4.0	51.30	88.10	34.80	
8.0	00.00	00.00	00.00	
His77 A				2.77
4.0	100.00	100.00	100.00	
8.0	00.00	00.00	00.00	
His77 B				2.77
4.0	100.00	00.00	100.00	
8.0	00.00	00.00	00.00	
His90 A				2.26
4.0	100.00	100.00	100.00	
8.0	00.00	00.00	00.00	
His90 B				2.26
4.0	100.00	100.00	96.70	
8.0	00.00	00.00	00.00	
Lys93 A				10.29
4.0	100.00	100.00	100.00	
8.0	100.00	100.00	100.00	
Lys93 B				10.29
4.0	100.00	100.00	100.00	
8.0	100.00	100.00	100.00	

Table 6. Residues with permanency time of hydrogen interactions (Hbond) of the *Aaeg*OBP1-ZINC71773878 complex under the effect of different pH during CpHMD. All values are given in %.

Residue	pH=8,0	pH=4,0
His77	0,05	14,27
Phe123	36,33	-

Table 7. Binding free energy between ZINC71773878 and cyclodextrins

Cyclodextrin complexed	Binding free energy (kJ/mol)
DMCD	-3.59
γ-CD	-3.26
α-CD	-2.92
β-CD	-3.52
TMCD	-3.62
Hp-β-CD	-3.43
RMCD	-3.60
SBE-β-CD	-3.66

Table 8. Prediction of storage stability of ZINC71773878 with the main cosmetic polymers available on the FormulationAI server.

Stabylity (90 days)					
Polymer	T = 25ºC	T = 37ºC	T = 40ºC	T = 45ºC	T = 50ºC
PEG8000	0,79	0,75	0,72	0,71	0,68
PEG2000	0,80	0,77	0,73	0,73	0,69
Stabylity (180 days)					
PEG8000	0,68	0,61	0,59	0,59	0,59
PEG2000	0,69	0,62	0,61	0,61	0,60

Table 9. Most potent compound of each cluster.

**Compound**	**Chemical sketch**	**K_i (μM)**

**01**	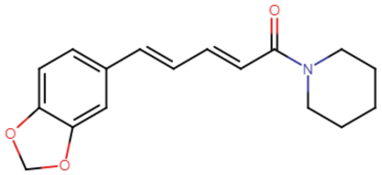	0.99
**02**	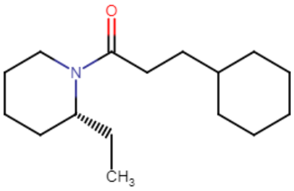	1.24
**05**	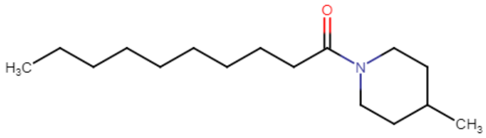	2.99
**11**	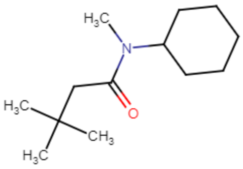	4.18
**13**	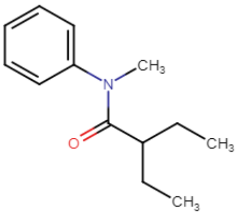	10.50
**21**	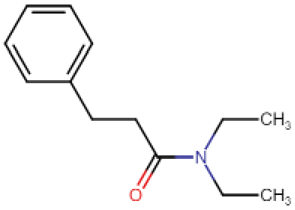	17.51

Table 10. Concentrations employed to perform the repellency assays.

Application sequence	Concentrations (µmol.mL^{-1})
Frist step	
Cage 01: Negative control	Ethanol
Cage 02: dosage 01	375
Cage 03: dosage 02	750
Cage 04: dosage 03	1500
Second step	
Cage 01: Negative control	Ethanol

Cage 02: dosage 04	2000
Cage 03: dosage 05	3000
Cage 04: Positive Control (DEET)	3000

Table 11. IUPAC name and chemical structure of compounds prioritized to molecular dynamics step

ZINC CODE	IUPAC name	Chemical sketch
ZINC380698	2-amino-N-(1,3-thiazol-2-yl)benzamide	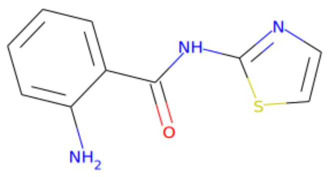
ZINC71773878	(2S)-2-(1,3-dioxoisoindol-2-yl)-3-methylbutanenitrile	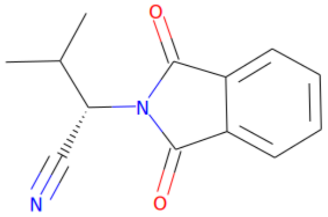
ZINC62702141	2-(4-oxo-3-prop-2-enylphthalazin-1-yl)acetic acid	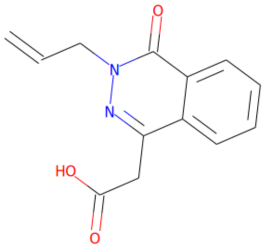
ZINC10483047	4-oxo-3-prop-2-enylphthalazine-1-carboxylic acid	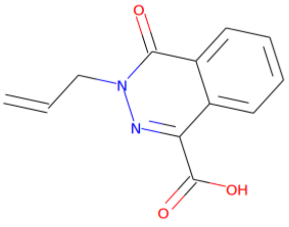

ZINC17917305	2-phenyl-3H-quinazolin-4-one	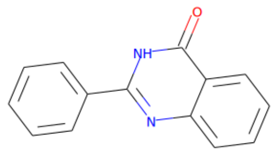
